# Biological Activities of p-Hydroxycinnamic Acids in Maintaining Gut Barrier Integrity and Function

**DOI:** 10.3390/foods12132636

**Published:** 2023-07-07

**Authors:** Zi-Ying Wang, Ying Yin, Dong-Ni Li, Dan-Yue Zhao, Jun-Qing Huang

**Affiliations:** 1 Interdisciplinary Institute for Personalized Medicine in Brain Disorders, School of Traditional Chinese Medicine, Jinan University, Guangzhou 510632, China; 2 Guangzhou Key Laboratory of Formula-Pattern of Traditional Chinese Medicine, Formula-Pattern Research Center, School of Traditional Chinese Medicine, Jinan University, Guangzhou 510632, China; 3 Department of Food Science and Nutrition, Faculty of Science, The Hong Kong Polytechnic University, Hong Kong SAR, China; daisydy.zhao@polyu.edu.hk; 4 Research Institute for Future Food, The Hong Kong Polytechnic University, Hong Kong SAR, China

**Keywords:** p-hydroxycinnamic acids, ferulic acid, caffeic acid, sinapic acid, p-coumaric acid, intestinal barrier

## Abstract

It is well established that p-Hydroxycinnamic acids (HCAs), including ferulic, caffeic, sinapic, and p-coumaric acids, possess a characteristic phenylpropanoid C6-C3 backbone and account for about one-third of the phenolic compounds in our diet. HCAs are typically associated with various plant cell wall components, including mono-, di-, and polysaccharides, sterols, polyamines, glycoproteins, and lignins. Interestingly, enzymes produced by intestinal microbes liberate HCAs from these associations. HCAs are completely absorbed in their free form upon ingestion and undergo specific reactions upon absorption in the small intestine or liver. The gut epithelium, composed of intestinal epithelial cells (IECs), acts as a physical barrier against harmful bacteria and a site for regulated interactions between bacteria and the gut lumen. Thus, maintaining the integrity of the epithelial barrier is essential for establishing a physiochemical environment conducive to homeostasis. This review summarizes the protective effects of HCAs on the intestinal barrier, achieved through four mechanisms: preserving tight junction proteins (TJPs), modulating pro-inflammatory cytokines, exerting antioxidant activity, and regulating the intestinal microbiota.

## 1. Introduction

Phenolic compounds are bioactive secondary metabolites found abundantly in plants [1]. Approximately 90% of polyphenols in the human diet are not absorbed in the small intestine but instead undergo decomposition and transformation by microorganisms residing in the colon [2]. These processes involve various biochemical reactions, leading to the generation of active molecules that can exert biological effects [3]. Intestinal polyphenols undergo a complex metabolic process that directly influences gut microbiota metabolism. This process encompasses biotransformation within epithelial cells, interactions with local microbiota, and conversion into conjugated derivatives, phenolic and aromatic acids, and other catabolites [4]. Notably, these small-molecular-weight metabolites have been shown to significantly contribute to the health-promoting properties of polyphenols [5].

**Figure 1:**
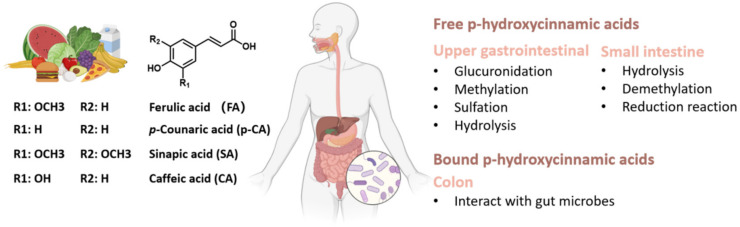
Structure of common p-hydroxycinnamic acids (HCAs), including ferulic acid (FA), p-coumaric acid (PCA), sinapic acid (SA), and caffeic acid (CA). The figure illustrates the absorption of HCAs and highlights the major metabolic reactions and organs involved in HCA metabolism.

Current evidence suggests that p-hydroxycinnamic acids (HCAs) are important natural phenolic compounds abundantly present in various foods, including fruits, vegetables, cereals, coffee, tea, and wine. These compounds are synthesized via the shikimate pathway and possess a phenylpropanoic acid C6-C3 core skeletal structure (Figure 1). 

Examples of HCAs include ferulic acid (FA), p-coumaric acid (PCA), sinapic acid (SA), and caffeic acid (CA). Although HCAs are widely distributed in cereals and herbal medicines, their impact on intestinal health has received significantly less attention than other polyphenols [6] primarily due to most of these compounds being bound to components such as mono-, di-, and polysaccharides, sterols, polyamines, glycoproteins, and lignins in plant cell walls. Humans lack the necessary enzymes to degrade plant sugars, such as pectin, xylan, and arabinose [7,8]. However, anaerobic microorganisms in the intestine ferment dietary fibers in association with different microbial species, resulting in the release of HCAs, as well as the production of short-chain fatty acids and gases, all of which contribute positively to the overall health of the host [9]. Thus, the interactions between the gut and HCAs are critical in maintaining host health.

The intestinal barrier is one of the most important biological barriers in the body and consists of various extracellular and cellular components [10]. The intestinal barrier acts as a semipermeable membrane that allows the passage of nutrients while preventing the entry of pathogens and harmful substances. Dysfunction of the intestinal barrier can lead to increased intestinal permeability and contribute to the development of serious diseases [10]. In this regard, the metabolism of phenolic compounds in the gut has been shown to optimize intestinal barrier function, which is beneficial for preventing and treating enteropathy and systemic disorders associated with barrier disruption, such as chronic metabolic diseases, liver diseases, and encephalopathy.

While several studies have investigated the direct biological effects of phenolic compounds on physiological functions, little attention has been attributed to the metabolism and physiological effects of HCAs following their ingestion into the gut. Therefore, this review sought to shed light on the bioactivities of HCAs on intestinal health and provide evidence regarding their impact on intestinal barrier function and the development of diseases associated with intestinal barrier dysfunction.

## 2. Metabolism of p-Hydroxycinnamic Acids in the Gut

### 2.1. Absorption and Metabolic Pathways of HCA

The absorption and bioavailability of pure HCAs in humans have not been extensively studied, mainly due to the complexity of individuals’ diets and the diverse food matrices consumed. Research suggests that dietary polyphenols, including HCAs, have limited absorption in the small intestine, with only approximately 10% of their absorption attributed to their interaction with other compounds present in plant cell walls, including mono-, di-, and polysaccharides, sterols, polyamines, glycoproteins, and lignins, which are components of plant cell walls. These polyphenols undergo microbial metabolism in the colon, producing metabolites such as phenylpropionic and benzoic acids [11]. Free HCAs, however, are absorbed intact throughout the gastrointestinal tract, while HCA esters and HCAs attached to carbohydrate chains are hydrolyzed by enzymes [12]. The absorption rate and extent of HCAs generally depend on their structure after ingestion. Studies have shown that the absorption capacity of bound HCAs by specific enterocytes in the gastrointestinal wall is lower than their free forms [13]. Additionally, during the process of intestinal absorption, HCAs can also modulate the bioavailability of other nutrients. For instance, FA has been found to inhibit the micellization and Caco-2 uptake of carotenoids. Therefore, when considering HCA intake, it is important to consider its potential impact on the digestion and absorption of other nutrients [14].

**Figure 2:**
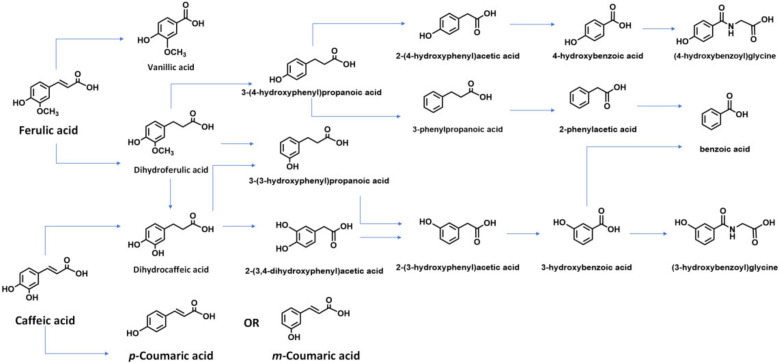
Major metabolites of HCAs under the biotransformation of gut microbes.

The small intestine serves as the initial site of HCA metabolism, where HCAs are hydrolyzed by esterase activity and other enzymes in mucosal cells. Subsequently, the colonic microflora becomes the main site of HCA hydrolysis. This includes the metabolites obtained from digestion in the small intestine, which occur independently of microbial digestion [15]. In the intestinal tract, most HCAs are transformed by microbial enzymes into small metabolites with or without bioactivities, which directly interact with the intestinal barrier. For instance, the esterase CaeA from Bifidobacterium longum subsp. longum can cleave several HCAs (Figure 2) [16]. Although the metabolism of HCAs in humans has not been fully elucidated, three key pathways have been identified: (1) absorption without transformation; (2) consumption in the stomach or small intestine, with or without hydrolysis, and further conjugation (sulfate, glucuronide, or methyl) or other forms of metabolism (hydrogenation, α- or β-oxidation, conjugation with glycine) [17]; and (3) conversion into later-stage metabolites mediated by colonic microflora, with or without further biotransformation [18]. The uptake and utilization of HCAs are highly dependent on their metabolism, with primary metabolism significantly influencing their bioavailability [19]. Enterocytes and liver cells can undergo glucose metabolism, sulfate incorporation, methylation, and hydrogenation. Additionally, HCAs can bind to glycine in the kidneys and liver and undergo demethylation and dehydrogenation in the liver [18].

### 2.2. Biotransformation of HCAs by Gut Microbes

It is now understood that most FAs are found in a conjugated form within plant cell walls. The proportion of bound ferulic acid can vary among different plant species. After ingestion, the hydrolytic activity of gut microbiota on ester-linked arabinoxylans leads to the production of free FA [20]. In the gut, microbial degradation of FA dimers results in the formation of vanillin and 3-(4-hydroxyphenyl)-propionic acid [20]. Feruloyl esterase EC 3.1.1.73 (FAE) from Lactobacillus acidophilus can induce the release of a small amount of free FA from the carbohydrate chain. Once in its free form, free FA can be metabolized into feruloylglycine, dihydro-FA, and its glucuronide/sulfate conjugates by colonic enzymes such as coenzyme A, reductases, and glucuronosyl transferase/sulfuryl-O-transferase, respectively. However, the gut microbes responsible for these enzymatic actions have not been fully identified. Interestingly, changes in the levels of endogenous metabolites in the host’s gut have been correlated with the effects on key enzymes and transporters involved in HCA biotransformation. In this respect, it has been shown that short-chain fatty acids (SCFAs) derived from gut microbes, such as propionate and butyrate, can enhance the transport of fatty acids and the appearance of FA glucuronide apically and basolaterally by upregulating the basolateral transporter (monocarboxylate transporter 1 (MCT1)) [21].

Bound p-CA in cell walls is conjugated with tartaric acid, glycerol, or glucose, making it difficult to be absorbed in the upper gastrointestinal tract. However, bound p-CA can be absorbed in the colon and released by cinnamoyl esterases produced by certain gut flora. An in vitro study confirmed that p-CA could be released from dietary fiber through human fecal co-fermentation [22]. The conjugates of p-CA experience a slower absorption rate than free p-CA and follow different pathways of biological transformation compared to free p-CA [23]. P-CA, which has a higher bioavailability than other HCAs, was reported to experience a higher absorption rate in the jejunum compared to other sections of the rat gastrointestinal tract [24]. 

In the small intestine, orally administered SA is absorbed through active Na^+^-gradient-driven transport [25]. Some studies have reported that the metabolism of SA is mediated by esterases from gut microflora [26], although the specific metabolic flora has not been identified. Metabolites of SA found in urine include sinapic acid, 3-hydroxy-5-methoxyphenylpropionic acid, methyl sinapate-sulfate, methyl sinapate-glucuronide, dihydrosinapic acid, 3-hydroxy-5-methoxycinnamic acid, and their acid-labile conjugates. These results indicate that free and ester forms of SA undergo phase I and II reactions in the human small intestinal epithelium, resulting in the formation of these metabolites [27].

Within the human intestinal tract, there is a synergistic interaction between various fecal microbes, such as *Gordonibacter urolithinfaciens* and *Ellagibacter isourolithinfaciens*. These microbes possess the ability to metabolize CA and its esters (chlorogenic acid (CGA) and rosmarinic acids (RA)) into 3-hydroxycinnamic acid and 3-hydroxyphenylpropionic acid (3-HPP) [28]. Subsequently, 3-HPP is dehydroxylated to form 3-phenylpropionic acid, further subjected to β-oxidation by gut microbiota, producing benzoic acid. Finally, benzoic acid undergoes glycination and is converted into the urinary metabolite hippuric acid [28]. 

Current evidence suggests that the colon or colonic microbiota primarily metabolize the majority of ingested CGA, a derivative of CA. Analysis of ileum fluid samples revealed that approximately 71% of ingested CGAs remain unmetabolized. Consistently, a study revealed that 77.5% of CGAs could be recovered from ileal fluids of ileostomized subjects after coffee intake [11]. Upon fermentation in the gut, the second metabolites of HCAs are absorbed and transferred throughout the body. Human intervention studies have examined the bioavailability of HCAs from food sources. Dihydro-derivatives of quinic acid were detected in plasma and urine, suggesting their synthesis or metabolism by gut microbiota from CGA [29]. An alternative bio-transformation pathway involves the conversion of CA and coenzyme A (CoA) into 3,4-dihydroxystyrene and 4-hydroxystyrene by tyrosine decarboxylase from *Enterococcus faecalis* [30]. The bioactivity of tyrosine decarboxylase was higher with CA compared to FA or CoA, based on the structure of HCAs. A proposed metabolic pathway of CGA in the human colon results in similar metabolites as CA. Gut flora can hydrolyze CGA into CA and quinic acids. Among the microorganisms speculated to drive this transformation in the human gut, *Escherichia coli* (three strains), *Lactobacillus gasseri* (two strains), and *Bifidobacterium animalis* have shown varying degrees of capability [31]. Specifically, *B. animalis subsp. animal-islactis* was identified to convert CGA into CA [32]. 

Intestinal microorganisms reportedly facilitate the degradation of rosmarinic acid, another derivative of CA, into simpler phenolic compounds. The absorption rate of RA was reported to be 59.14%, but its intestinal permeability is much lower, accounting for approximately 1% of the applied dose [33]. Consequently, the compound’s absorption occurs through enterocytes via paracellular transport, facilitated by tight junctions [34]. An in vitro study found that RA from *Thymus vulgaris* undergoes a rapid decline within the first 6 h of fermentation, and its concentration is reduced to trace levels after 12 h. Furthermore, content analysis of human feces from individuals consuming thyme-enriched olive oil for over 3 weeks revealed the absence of RA [35]. In addition, over 90% RA can be hydrolyzed by probiotic Lactobacillus strains [36]. These clinical studies collectively demonstrate that the significant degradation of HCAs in the gastrointestinal tract depends on gut microbes.

## 3. Protective Effects of HCAs on the Intestinal Barrier

### 3.1. Physiological Function and Composition of Intestinal Barrier

#### 3.1.1. Structure of Intestinal Barrier

Importantly, IECs establish barriers between the external environment and the body’s internal environment. Preserving the integrity of the intestinal barrier is of utmost importance in preventing the entry of harmful substances, viruses, and microorganisms into the gut. Disruption of the outer mucus layer and the epithelium can compromise the inner gut vascular barrier, resulting in the systemic dissemination of microbes or microbial-derived molecules. Gut dysbiosis has been associated with the early stages of various extraintestinal pathologies characterized by gut vascular barrier leakage. Subsequent disruption of the vascular barriers at different sites facilitates the propagation of inflammatory signals to distant organs, highlighting the interconnectedness of the anatomical obstacles throughout the body. This phenomenon also contributes to the functional and pathological interactions mediated by the brain–gut and liver–gut axes [37].

The gut–liver axis refers to the bidirectional relationship between the gut microbiota and the liver [38]. This relationship results from the integration of signals generated by dietary, genetic, and environmental factors. The portal vein establishes this reciprocal interaction by enabling the transport of gut-derived products directly to the liver, while the liver provides feedback through bile and antibody secretion to the intestine. The gut–brain axis is the complex, bidirectional communication system between the central nervous system (CNS) and the gastrointestinal tract [39]. It is a pathway through which the gut and the brain send signals to each other, influencing processes such as digestion, mood, mental health, and overall well-being. This communication system involves various mechanisms, including neural, hormonal, and immune pathways. It plays a crucial role in maintaining homeostasis and is increasingly recognized for contributing to multiple diseases, including neurological disorders, mental health conditions, and gastrointestinal diseases [40].

**Figure 3:**
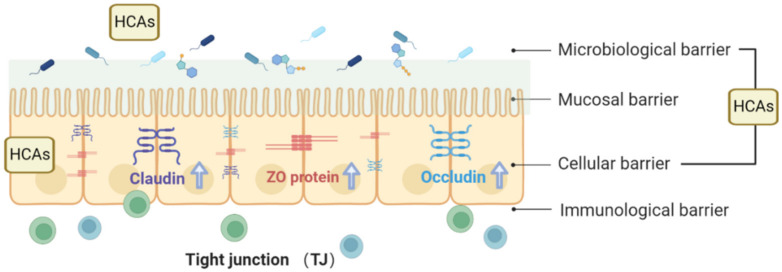
HCAs exert a protective effect on the intestinal barrier by facilitating the assembly of epithelial tight junctions and promoting the abundance of occludin, claudin-1, and ZO-1 proteins.

The intestinal barrier can be divided into four layers: the microbiological barrier, the mucosal barrier, the cellular barrier, and the immunological barrier (Figure 3) [10]. The microbiological barrier is located in the outermost position of the mucus layer and is formed by the presence of microorganisms and their metabolites. The mucosal barrier consists primarily of macromolecules, including proteins, enzymes, peptides, and immunoglobulins. The cellular barrier lies beneath the mucus layer and comprises IECs. These cells serve as the source of the mucosal barrier, with goblet cells secreting Mucin2 (MUC2), the main mucus protein that forms an essential component of the mucus layer. The immunological barrier, located beneath the intestinal epithelium, comprises various immune cells such as T lymphocytes, B lymphocytes, dendritic cells, macrophages, and plasma cells. This barrier is critical in innate and adaptive immune responses, including antigen presentation, secretion of inflammatory mediators, and antibody production [41]. In recent years, numerous studies have focused on the interaction between HCAs and the intestinal barrier, particularly the cellular barrier.

Beneath the mucosal barrier, the cellular barrier is composed of a continuous layer of IECs, which includes normal epithelial cells and several types of cells with specific functions, including enteroendocrine cells, goblet cells, enterocytes, Paneth cells, and micro-fold cells [42]. Enteroendocrine cells reportedly play a crucial role in connecting the central and enteric neuroendocrine systems through the secretion of numerous hormones that regulate digestive function. Conversely, Paneth cells secrete antibacterial peptides, such as lysozyme and defensins, which prevent the colonization of harmful bacteria [43]. Goblet cells maintain the mucosal layer by secreting mucin, a highly glycosylated protein that forms an extensive net-like structure [44]. The secretion of mucins by goblet cells into the intestinal lumen is the first line of defense against microbes. Among these mucins, MUC2 is the most abundant and plays a crucial role in epithelial restitution and keeping intestinal microbes at a distance from the epithelial surface [45]. Components of the normal microbiota directly contribute to the intestine’s barrier function by inducing mucin production and secretion by goblet cells [46].

#### 3.1.2. Physiological Functions of TJs

The apical junctional complex consists of desmosomes, adherens junctions, and TJs, which adhere to adjacent epithelial cells and regulate the paracellular movement of solutes and ions (Figure 3). Desmosomes and adherens junctions primarily serve as physical attachments between cells, while TJs function as selective, semipermeable barriers to the intercellular space [47]. TJs are multifunctional complexes composed of transmembrane and cytosolic proteins, including occludin, claudins, zonula occludens (ZOs), tricellulin, cingulin, and junctional adhesion molecules (JAMs) [48]. Among these, claudin proteins are considered the structural backbone of TJs and regulate the selectivity of the epithelial barrier by forming charge- and size-specific channels between epithelial cells. In the intestine, claudins-1, 3, 4, 5, and 8 provide essential TJ sealing to decrease paracellular permeability, while claudin-2 forms charge-selective pores. The colon, in particular, exhibits a high expression of claudins-1, 3, 4, 5, and 8, while claudins-2, 7, and 12 contribute to mediating intestinal permeability [49]. 

It has been established that occludin, claudin, and tricellulin link adjacent cells to the actin cytoskeleton through intracellular tight junction proteins, such as ZO proteins, which are crucial regulators of tight junction permeability that act on apical receptors to increase permeability and facilitate absorption. Luminal factors, such as food and bacterial toxins, can release ZOs, which connect tight junction transmembrane proteins to cytoskeletal complexes [50]. Adherens junctions below TJs mainly consist of e-cadherin, catenin, and actin filaments. These protein complexes contribute to cell–cell signaling, epithelial restitution, and the stability of desmosomes, supporting epithelial stability [51]. 

Furthermore, cytokines such as tumor necrosis factor-α (TNFα), interferon-γ (IFN-γ), and interleukins play a crucial role in regulating tight junction integrity [52]. Tight junction proteins activate various cellular processes to maintain the integrity of the intestinal barrier through their composition and interactions within the complex. TJs are essential for maintaining intestinal permeability by regulating paracellular flux and epithelial permeability [53]. As gatekeepers, they impede the ingress of foreign antigens, thereby mitigating inflammation and potential tissue damage. TJs also serve as sites for epithelial interaction with cells such as dendritic cells (DCs), allowing them to sample external antigens [54]. Recent reports investigated the barrier function of the epithelium in various immune-related diseases. Thus, maintaining the function of TJs is essential for preventing and managing inflammatory and autoimmune diseases [55].

### 3.2. Biological Activities of HCAs on the Intestinal Barrier

#### 3.2.1. HCAs Modulation of Intestinal Permeability by Protecting TJs

HCAs have been shown to modulate intestinal permeability and exert protective effects on the intestinal barrier. The intestinal tract acts as a boundary to prevent the invasion of harmful molecules into the mucosal tissue and systemic circulation. Increased intestinal permeability has been documented in various diseases, including intestinal inflammatory diseases, associated with systemic inflammation and altered gut immune function. In metabolic diseases, studies pointed out specific roles of the intestinal immune system and IECs. When the finely tuned zonulin pathway is dysregulated in genetically susceptible individuals, intestinal and extraintestinal autoimmune, inflammatory, and neoplastic disorders can occur [50,56]. In addition, the beneficial effects of HCAs have been established in modulating intestinal permeability and protecting against multifactorial diseases.

FA has been found to inhibit the decrease in occludin and ZO-1 mRNA and protein expression caused by lipopolysaccharide (LPS) and stimulate the expression of miR-200c-3p. A study revealed that in Caco-2 cells, FA promotes the activation of the phosphatidylinositol-3 kinase (PI3K)/protein kinase B (AKT) pathway by suppressing the negative regulator phosphatase and tensin homolog (PTEN) via miR-200c-3p, which maintains the function of tight junctions and ameliorates LPS-induced epithelial barrier dysfunction [57]. Another HCA, SA, has been reported to reverse the redistribution of the ZO-1 and claudin-1 proteins in LPS-treated Caco-2 cells and attenuate epithelial permeability. SA has been reported to reduce the activation of the Myosin light-chain kinase (MLC) pathway, which plays essential roles in cellular functions such as cell morphogenesis, motility, and smooth muscle contraction [58]. In a colitis mouse model, SA was found to enhance the expression of ZO-1, occludin, and claudin-1 [59]. 

CGA, a derivative of CA, has been shown to prevent MLC phosphorylation by inhibiting MLC activation and increasing F-actin polymerization in the cytoplasm. CGA also inhibits the activation of rho-associated coiled-coil-containing kinases 1 (ROCK1) signals [60]. In colitis rats, CGA promotes the assembly of epithelial tight junctions by decreasing the phosphorylation of occludin and claudin-1, thereby reducing intestinal permeability and attenuating intestinal barrier damage. Dietary supplementation of CGA increases the abundance of intestinal occludin and ZO-1 proteins, ameliorating intestinal mucosal injury and protecting the intestinal barrier in LPS-challenged rats. Similarly, FA significantly restores the expression of occludin, ZO-1, and E-cadherin in heat-stress-induced intestinal epithelial barrier dysfunction, indicating its potential protective effect [61]. Furthermore, CGA reduces high-fat-diet (HFD)-induced weight gain and adiposity, improves the intestinal barrier, and prevents metabolic endotoxemia (*p* < 0.05). In summary, HCAs exert protective effects on the intestinal barrier by promoting the assembly of epithelial tight junctions and increasing the abundance of occludin, claudin-1, and ZO-1 proteins (Figure 3). HCAs, including FA, salvianolic acid, and CGA, act on various cellular pathways to enhance the integrity of tight junctions.

#### 3.2.2. Modulatory Effects of HCAs on Intestinal Immunity

The intestinal immune system promotes mucosal immunity and facilitates tolerance toward dietary and microbial antigens. It comprises innate and adaptive components within the intestinal epithelia and lamina propria [62]. In addition to its role as a physical barrier, the intestinal epithelium secretes various molecules that contribute to maintaining intestinal homeostasis. Epithelial cells secrete cytokines and chemokines that regulate the chemotaxis of immune cells, including neutrophils, macrophages, basophils, and T cells. The epithelium plays a key role in modulating immune cell function and activation [63]. Pro-inflammatory chemokines, such as IL-8, are involved in inflammatory bowel disease pathogenesis and neutrophil infiltration [64]. Interestingly, HCAs have been shown to inhibit pro-inflammatory chemokines and exhibit beneficial effects in various intestinal diseases.

For example, feruloylated oligosaccharides derived from maize bran, which are esters of FA and arabinose xylose, have been shown to alleviate colitis induced by dextran sulfate sodium and exert potent immune modulatory effects by restoring the immune balance of Th17 and Treg cells [65]. FA treatment was also found to increase the number of granulocytes in mesenteric lymph nodes, the number of spleen monocytes/granulocytes, and plasma levels of interleukin-2 (IL-2) and interleukin-12 (IL-12) in mice [66].

In allergic asthma, SA has been found to ameliorate airway inflammation by suppressing T-helper 2 immune responses [58]. The regulatory effect of SA on immunity may be associated with its anti-inflammatory effects. It has been shown to reduce the inflammatory response in colitis mice by inhibiting the Toll-like receptor 4 (TLR4)/Nuclear factor kappa B (NF-κB) pathway and NLR-family-pyrin-domain-containing 3 (NLRP3) inflammasome activation [59]. SA also effectively ameliorated ulcerative colitis in rats by suppressing TNF-α, interleukin 6 (IL-6), Myeloperoxidase (MPO), Prostaglandin E2 (PGE2), cyclooxygenase-2 (COX-2), and NF-κB [67].

Although there is currently no direct evidence of immune modulation by caffeic acid, it has been suggested that CA may regulate intestinal immunity through its anti-inflammatory effects. Molecular mechanism analysis has revealed that CA significantly inhibits the mRNA levels of Caspase-1 and NLRP3 and is absent in melanoma 2 (AIM2) in radiation-induced intestinal injury [68]. Additionally, CA was found to counter the formation of PGE2 and reduce interleukin 8 (IL-8) in human intestinal cells treated with the pro-inflammatory cytokines IL-1β. In addition, the release of pro-inflammatory cytokines TNF-α, IL-6, and INF-γ was inhibited by interfering with the NF-κB pathway, which has been associated with intestinal inflammatory signals such as intestinal epithelial cell apoptosis and leukocyte migration [69]. Recent research has demonstrated that CA and p-CA activate the focal adhesion kinase (FAK)-phosphatidylinositol 3-kinase (PI3K)-Akt signaling pathway and protect against LPS-injured intestinal epithelial cell line 6 (IEC-6) cells [70].

In hyperuricemia mice, CGA significantly reduced serum LPS levels and the mRNA expression of IL-1β, TNF-α, NOD-like receptor NLRP3, and caspase-1. It inhibited the activation of the myeloid differentiation factor 88 TLR4/MyD88 (myeloid differentiation factor 88)/NF-κB signaling pathway in the kidney, relieving inflammation in hyperuricemia mice [29]. CGA also significantly inhibits the release of pro-inflammatory cytokines, such as MPO, in models of dextran sodium sulfate (DSS)-induced colonic injury, thereby protecting the intestinal barrier [71]. Another study revealed that CGA treatment reduces the concentrations of pro-inflammatory cytokines IL-6 and IL-8, as well as the levels of COX-2 and TNF-α mRNA compared to LPS-treated cells. Similarly, studies have shown that CGA attenuates oxidative-stress-induced intestinal epithelial inflammation and injury by co-regulating PI3K/Akt and inflammatory signals such as intestinal epithelial cell apoptosis. The mechanism of CGA in alleviating ulcerative colitis involves the mitogen-activated protein kinase (MAPK)/extracellular-signal-regulated kinase (ERK)/Jun N-terminal kinase (JNK) signaling pathway, as it decreases the expression of ERK1/2, p-ERK, p38, p-p38, JNK, and p-JNK proteins, ultimately inhibiting DSS-induced colitis, oxidative stress, and apoptosis [72]. Additionally, CGA has been demonstrated to exert beneficial effects on alleviating oxidative stress and inflammatory responses while improving intestinal barrier health [18].

#### 3.2.3. Antioxidant Properties and Benefits for the Intestinal Barrier

Oxidative stress occurs when there is an imbalance between generating and eliminating reactive oxygen species (ROS). Current evidence suggests that increased cellular ROS leads to reactions with proteins, fats, and DNA and initiates related signaling pathways. Prolonged exposure to ROS and an imbalance in their production can contribute to various intestinal diseases [73]. Intestinal oxidative stress plays a critical role in the early stages of intestinal injury, and maintaining a balance of ROS is essential for treating intestinal diseases [74]. Several studies have revealed the protective effects of HCAs and identified related mechanisms that can relieve oxidative stress in intestinal diseases.

FA has been shown to inhibit ROS production and aldose reductase activity, activate the PI3K/Akt signaling pathway, and exhibit an antioxidant role through nuclear-factor-erythroid-2-related factor 2 (Nrf2)/Heme oxygenase-1 (HO-1) pathway activation, thereby enhancing its overall antioxidant effect [75]. In experiments where cells were pretreated with FA before exposure to H_2_O_2_, FA increased the levels of catalase [35] and superoxide dismutase (SOD) while decreasing the levels of MDA and ROS. FA also inhibited the decrease in mitochondrial membrane potential, protecting cells from H_2_O_2_-induced damage and improving cell survival by regulating intracellular antioxidant enzyme activity and cell cycle distribution [76]. In addition, FA supplementation in piglets resulted in improved feed conversion ratio, increased serum total superoxide dismutase and catalase activities, elevated lactase and maltase activities in the duodenum, and increased jejunal villus height and maltase activity. It reduced the duodenal crypt depth and duodenal and jejunal cell apoptosis, and cleaved caspase-3 and caspase-9 contents [77]. Additionally, dietary FA supplementation increased the activities of CAT, T-SOD, and glutathione peroxidase (GSH-Px), as well as the mRNA levels of SOD1, SOD2, catalase (CAT), Glutathione S-transferases (GSTs), glutathione peroxidase 1 (GPX1), glutathione reductase (GR), Nrf2, hormone-sensitive lipase (HSL), carnitine palmitoyltransferase-1b (CPT1b), and peroxisome-proliferator-activated receptor α (PPARα) in piglets, while reducing the contents of MDA and triglyceride (TG). It also upregulated protein levels of Nrf2, HO-1, and NAD(P)H quinone oxidoreductase 1 (NQO1) in the longissimus dorsi muscle [78]. FA has also been suggested to inhibit free radical formation, lipid peroxidation, and DNA damage, acting as a radical scavenger and antioxidant. For example, in sepsis-induced DNA damage, FA neutralizes the toxic effects of ROS generated by sepsis, reduces DNA damage, and improves the body’s antioxidant status [78]. Oral administration of feruloylated oligosaccharides derived from maize bran exhibited antioxidant effects in the plasma, liver, kidney, and heart of diabetic rats and had inhibitory effects on the formation of advanced glycation end products (AGEs) and N-ε-(carboxymethyl) lysine (CML) in the organs [79].

SA was found to significantly ameliorate colonic injuries in AA-induced UC by suppressing MDA and nitric oxide (NO) levels and restoring the oxidant balance indicated by catalase and glutathione levels. In a high-fat-diet rat model, SA administration effectively reduced ROS and MDA in the colon and increased liver total antioxidant capacity. SA also alleviated oxidative stress in the liver and colon, lowered TG and non-esterified fatty acid (NEFA) levels, and inhibited the growth of bacterial species associated with disease and inflammation [80].

Studies have shown that CA and CGA protect against intestinal ischemia–reperfusion (I/R) injury in the rat small intestine. In this respect, CA supplementation enhanced antioxidant capacity and effectively alleviated DSS-induced colitis in mice, which may be related to the activation of the Nrf-2/HO-1 pathway and increased mRNA expression of Nrf-2, HO-1, and NQO1 [81]. CA attenuated oxidative DNA damage; reduced cytotoxicity, protein, and lipid peroxidation; and restored antioxidant capacity. These beneficial effects are mediated by the augmentation of reduced glutathione (GSH) and effective ROS scavenging by CA [82]. In the colon, CA phenethyl ester always activated Nrf2, followed by increased HO-1 and NQO-1 levels and downregulated nuclear levels of p65 and c-Jun, which suppressed DSS-induced colonic oxidative stress [83].

CGA has also been demonstrated to have beneficial effects on improving the capacity of antioxidant enzymes such as SOD, CAT, peroxidase (POX), GST, and glutathione peroxidase (GPX), thereby effectively preventing ROS accumulation and lipid peroxidation [84]. In a non-alcoholic fatty liver disease (NAFLD) model, CGA increased the expression of tight junction proteins occludin and ZO-1 in intestinal tissue, decreased the LPS levels, and increased the level of glucagon-like peptide 1 (GLP-1) in the portal vein [85].

#### 3.2.4. Regulatory Effects of HCAs on Gut Microbiota

The intestinal region of mammals is colonized by many microorganisms containing trillions of bacteria collectively referred to as the gut microbiota [85]. Symbiotic bacteria have been identified to provide the host with several functions that enhance immune homeostasis and immune responses and protect against pathogen colonization. Consequently, targeting the modulation of gut microbiota has emerged as a therapeutic approach for treating intestinal diseases.

A study revealed that dietary supplementation of CA could enhance the anti-inflammatory and antioxidative capacity by modulating the gut microbiota community and improving gut barrier function in mice with DSS-induced colitis [81]. *Akkermansia*, crucial for maintaining the integrity of the mucus layer in obese patients, also inhibits inflammatory cytokines and low-density lipoprotein (LDL) pathways associated with lipid accumulation. CA can potentially restore microbial richness and alleviate colonic pathology and inflammation in DSS colitis mice, which may be linked to increased *Akkermansia* abundance [86]. Furthermore, research has shown that 2% CGA can enhance microbial diversity in the colon, particularly increasing the relative abundances of *Akkermansia* and *Lactobacillus* and potentially rectifying the microecological disorder induced by DSS [87]. Studies have established a connection between dietary CGA and the intestinal microbiota, demonstrating that CGA supplementation has beneficial effects on improving intestinal function, regulating the abundance of specific bacteria in the cecum of weaned pigs, enhancing intestinal antioxidant capacity, and positively influencing gastrointestinal development [88]. In the same DSS-induced colitis model, CGA was found to modulate the gut microbial community structure, leading to reduced intestinal and systemic inflammation and improving the course of DSS-induced colitis [89]. Moreover, CGA increased the relative abundance of SCFA-producing bacteria, including *Bacillus* spp., *Pseudomonas* spp *UGC-001*, and *S. butyricum*, while also reversing the purine and glutamate metabolism functions of the intestinal flora [90]. In models of DSS-induced colonic injury, CGA has been demonstrated to restore intestinal microbial diversity by increasing the relative abundance of *Akkermansia* and *Lactobacillus*, resulting in reduced DSS-induced intestinal injury and intestinal barrier protection [81].

Numerous studies have reported the relationship between HCAs and gut microbes in relation to obesity. A recent study discovered that CA consumption led to decreased body weight and fat accumulation, improved lipid profiles, and increased energy expenditure in diet-induced obese (DIO) mice. Furthermore, CA was found to restore gut microbiota homeostasis and increase the prevalence of anti-obesity and butyrate-producing bacteria in these mice [91]. In addition to CA, the protective effect of CGA against obesity relies on its regulation of gut microbiota structure, diversity, and changes in relative abundance at various taxonomic levels, from phylum to genus [92]. Moreover, a study demonstrated that 0.25% or 0.5% dietary FA ameliorated lipogenesis and fat deposition in white adipose tissue (WAT) and lipid accumulation in the liver of obese C57BL/6J-ob/ob mice [93].

Experiments involving human feces have shown that tea and coffee intake may benefit the intestinal environment by modulating the population of intestinal bacteria [94]. Indeed, HCAs have the potential to beneficially impact gut microbiota, and their biotransformation products significantly promote the growth of Bifidobacterium spp. and modulate the Firmicutes/Bacteroidetes ratio, an important indicator for assessing intestinal health [95]. The role of HCAs in other aspects of the body has been linked to the intestinal microbe. FA can potentially ameliorate NAFLD, which may be closely related to lipid and fatty acid metabolism via the remodeling of gut microbiota [96]. Additionally, SA effectively attenuated oxidative stress in the liver and colon, reduced plasma triglyceride and non-esterified fatty acid levels, and improved the diversity of the gut microbiota, mitigating HFD-induced intestinal dysbiosis [80]. Furthermore, SA supplementation significantly impacted the intestinal microbiome at various taxonomic levels by inhibiting the growth of bacterial species associated with diseases and inflammation, such as Bacteroides and *Desulfovibrionaceaesp*. These findings contribute to a better understanding of the effects of HCAs on human health and nutrition [80] (Table 1).

## 4. Conclusions

Overall, the above findings suggest that HCAs play a protective role in maintaining the integrity of the intestinal barrier. They promote the assembly of epithelial tight junctions and increase the abundance of occludin and ZO-1 proteins, thereby reducing intestinal permeability and mitigating damage to the intestinal mucosa caused by compromised tight junctions. HCAs have also been shown to decrease the levels of pro-inflammatory cytokines IL-6 and IL-8 and the mRNA expression of COX-2 and TNF-α.

Furthermore, HCAs can alleviate oxidative stress and inflammatory responses, improve intestinal barrier health, reduce inflammation, and regulate intestinal homeostasis. Animal experiments have demonstrated that supplementing HCAs effectively alleviates DSS-induced colitis in mice by enhancing the intestinal barrier, modulating intestinal microbial homeostasis, and boosting anti-inflammatory and antioxidant capacity. HCAs act as free radical scavengers and antioxidants by increasing the levels of CAT and SOD, reducing the levels of MDA and ROS, and inhibiting free radical formation, lipid peroxidation, and DNA damage.

Moreover, HCAs contribute to the restoration of intestinal microbial diversity, leading to a reduction in DSS-induced intestinal damage and protection of the intestinal barrier. HCAs can potentially exert beneficial effects on the intestinal microbiota, and their biotransformation products significantly promote a healthy intestinal flora ecology.

In summary, HCAs yield a favorable impact on the protection and restoration of the intestinal barrier through various mechanisms: (1) modulation of intestinal permeability by safeguarding tight junctions, (2) modulation of intestinal immunity, (3) antioxidant properties that benefit the intestinal barrier, and (4) regulation of gut microbiota by HCAs.

## Figures and Tables

**Table 1:** Overview of underlying mechanisms of major HCAs/metabolites against intestinal disease (↑ means increase, ↓ means decrease).

Compounds	Models	Dose and Time	Mechanisms	Reference
Ferulic acid (FA)	LPS-induced Caco-2 cells	25, 50, 100 μM for 2 h	occludin, ZO-1 mRNA↑, PI3K/AKT↑, miR-200c-3p ↑,	[57]
	LPS in intestinal Caco-2 cell	1 μM for 2, 6, 48 h	Nrf2↑, NF-κB, p38 MAPK ↓	[97]
	Heat-stress-induced IEC-6 cells	5, 10, 20 μM for 4 h	PI3K/Akt↑, Nrf2/HO-1 ↑	[98]
	H_2_O_2_-induced cell death	5 μM for 24 h	CAT and SOD↑, MDA and ROS↓, mitochondrial membrane potential ↑	[76]
	Weaned piglets	0.05% and 0.45% dietary supplementation for 5 weeks	Nrf2-ARE↑, T-SOD, CAT, and GSH-Px ↑, MDA ↓	[78]
	male ApoE−/− mice	30 mg/kg/day of FA for 12 weeks	*Bacteroidetes*↑, *Firmicutes*↓	[99]
	Male C57BL/6J-ob/ob mice	0.05% to 0.5% *w*/*w* pure FA diet	*Firmicutes, Bacteroidetes, Dubosiella, Negativibacillus*↓, *Proteobacteria*↑	[93]
Sinapic acid (SA)	LPS-induced Caco-2 cells	5, 10, or 15 μmol/L for 24 h	MLC pathway ↓	[58]
			TLR4/NF-κB↓	
	DSS-induced colitis mice	10, 50 mg/kg for 7 days	ZO-1, claudin-1 ↑	[59]
			NLRP3 inflammasome ↓	
	Acetic-acid-induced ulcerative colitis rat model	40 mg/kg/day for 7 days	TNF-α↓, IL-6↓, MPO, PGE2, COX-2, and NF-κB ↑	[67]
			MDA, NO↓, restoring oxidant balance	
	High-fat-diet rat model	200 mg/kg for 8 weeks	ROS and MDA ↓	[100]
			*Roseburia*, *Lachnospiraceae*, *Blautia*, *Dorea* ↑	
			*Proteobacteria*, *Bacteroides*, *Desulfovibrionaceaesp* ↓	
	DSS-induced colitis in mice	251 mg/kg (*w*/*w*) CA for 23 days	Nrf-2/HO-1↑, mRNA expression of Nrf-2, HO-1, and NQO1↑	[81]
			mRNA expression of IL-1β, IL-6, and TNF-α ↓, SOD1, GPX1, GPX2, CAT, and IL-10↑	
			*Bacteroides* and *Turicibacter*↓, *Alistipes* and *Dubosiella* ↑, *Dubosiella* and *Akkermansia*↑	
	Indomethacin-induced and diclofenac-induced Caco-2 cell death	100 μM for 72 h	reducing intracellular ROS formation and altered mitochondrial transmembrane potential	[101]
	High-fat-diet-induced mice	35 mg/kg for 12 weeks	IL-1β, IL-6, MCP-1, TNF-α, and Nrf2 ↓, MMP-2 activity ↓	[102]
	DSS colitis mice	1.8 mg/kg/day	*Akkermansia*↑, ratio of *Firmicute to Bacteroidetes*↑	[86]
			IL-6, TNFα, and IFNγ↓, NF-κB activation ↓	
Chlorogenic acid (CGA)	H_2_O_2_-induced cell death	0.5, 1, 2 mmol/L for 3 h	IL-8, TNFα↓	[103]
	DSS-induced colitis mice	1 mM for 15 days	mRNA expression of colonic macrophage inflammatory protein 2 and IL-1β ↑	
	DSS-induced colitis mice	30, 60, 120 mg/kg for 10 days	ERK1/2, p-ERK, p38, p-p38, JNK, and p-JNK↓	[72]
			IL-1β, IL-6, TNF-α, PAF, PGE2↓, MPO, and IL-10↓	
	DSS-induced colitis mice	100, 200 mg/kg for 4 days	ERK1/2, JNK1/2, p-AKT, and p-STAT3 ↓	[104]
	PMA- and IFNγ-induced Caco-2 cells	0.2, 1, and 2 mM for 24 h	ROS↓, GSH↑, and Nrf2 ↑	[105]
	Twenty-four weaned pigs	1000 mg/kg	*Firmicutes, Bacteroidetes, Lactobacillus* ↑, *Proteobacteria* ↓, *Bifidobacterium, Lactobacillus* ↑	[88]

## Data Availability

Data is contained within the article.
